# Investigating the influence of drone flight on the stability of cancer medicines

**DOI:** 10.1371/journal.pone.0278873

**Published:** 2023-01-06

**Authors:** Wanqing Zhu, Andy Oakey, Paul G. Royall, Tim P. Waters, Tom Cherrett, Katherine Theobald, Ans-Mari Bester, Robert Lucas

**Affiliations:** 1 Pharmacy Department, Faculty of Life Sciences & Medicine, King’s College London, London, United Kingdom; 2 Transportation Research Group, Faculty of Engineering and Physical Sciences, University of Southampton, Southampton, United Kingdom; 3 Institute of Sound and Vibration Research, Faculty of Engineering and Physical Sciences, University of Southampton, Southampton, United Kingdom; 4 St. Mary’s Hospital, Isle of Wight NHS Trust, Newport, United Kingdom; 5 Portsmouth Pharmacy Manufacturing Unit, Portsmouth Hospitals NHS Trust, Portsmouth, United Kingdom; Federal University of ABC, BRAZIL

## Abstract

Monoclonal Antibodies (mAbs) are being used in the treatment of both malignant and non-malignant diseases and whilst highly effective, certain products have very short expiry times. Clinical deterioration and supply chain disruption can often lead to wastage and there is a need to reduce this by improving efficiency in logistics practices between manufacturing sites and administration locations. This study aimed to investigate the influence of drone flight on the stability of cancer medicines. Clinically expired, premanufactured monoclonal antibodies (mAbs) were investigated, contained inside instrumented Versapaks, and flown in a Skylift (Mugin) V50 vertical take-off and landing drone through seven phases of flight, (take-off, hover, transition, cruise, transition, hover, and landing). Storage specifications (2–8°C) were met, and any vibrations emanating from the drone and transmitted through the packaging during flight were monitored using accelerometers. Vibration occurred largely above 44 Hz which was consistent with rotor speeds during operation and was significantly greater in amplitude during transition than in forward flight or in hover. Bench experiments validated assurance practices, exploring the edge-of-quality failure by applying extremes of rotational vibration to the mAbs. Aggregation and fragmentation represented a loss of quality in the mAbs and would pose a risk to patient safety. No significant difference was identified in the aggregation and fragmentation of all flown mAbs samples, indicating structural integrity. Flown mAbs in their infusion bags had similar particle sizes compared to controls, (Bevacizumab 11.8±0.17 nm vs. 11.6±0.05 nm, Trastuzumab 11.2±0.05 nm vs. 11.3±0.13 nm, Rituximab 11.4±0.27 nm vs. 11.5±0.05 nm) and aggregate content (Bevacizumab 1.25±0.03% vs 1.32±0.02% p = 0.11, Trastuzumab 0.15±0.06% vs. 0.16±0.06% p = 0.75, Rituximab 0.11±0.02% vs. 0.11±0.01% p = 0.73). The quality of the three mAbs was assured, suggesting that the V50 drone did not induce sufficient levels of vibration to adversely affect their quality.

## 1. Introduction

Production of intravenously administered medicines costs £3.8 billion or approximately 3% of the National Health Service (NHS) England’s total annual budget [1]. Pharmacy aseptic services prepare sterile medicines such as chemotherapies for patients based on a national dose banding table that takes into account their height and weight [2]. These valuable treatments typically have a short shelf-life (often measured in hours) and require timely and reactive logistics services in order to meet patients’ needs [3]. This is particularly the case with Monoclonal Antibodies (mAbs) which are now an integral part in the treatment of haematology and non-haematology diseases and are bespoke made according to the individual patient’s needs [4].

Where the administration point for the treatment may be at a distance from the manufacturing unit, any inefficiencies in the logistics system can lead to strict time windows for treatment being missed and medicine wastage [5, 6]. In the case of St Mary’s Hospital on the Isle of Wight (IOW, UK), deliveries of mAbs are mainly received from a manufacturer in Portsmouth (mainland UK) where wastage rates of delivered treatments of approximately 7% of delivered treatments in a measured month were observed, with a typical value of £1,860 per treatment [7, 8]. Changes to patient treatment plans can also lead to mAbs being wasted [9], and with this in mind, a more responsive and rapid method of transportation involving aerial drones could, under certain circumstances, enable greater flexibility and reliability. Changes in the way treatments are timetabled may also assist with reducing wastage, in terms of better aligning the ordering process with administration appointments and wider inventory management [10].

There are strict carriage requirements for the transportation of aseptic medicines set out by the UK’s Medicines and Healthcare Products Regulatory Agency (MHRA) to ensure product quality is maintained [11]. Following good distribution practice, these regulations stipulate that multi-layer packaging must be used and product temperatures must be maintained, typically using cool packs and approved carriage containers [12, 13].

For certain medical cargos, the control of temperature during the transportation process is very important and has been the key focus of attention in terms of good practice in carriage requirements, however, transportation also generates vibrational perturbations [14, 15]. For commercial road vehicles, vibration is well below 100 Hz and typically less than 1 g rms (root mean square) in the vertical direction, although values as high as 3.4 g have been reported in literature [16]. Vibration levels in copter-style drones can be significantly higher and can occur predominantly above 100 Hz [17]. Before drones can be considered as a viable mode, the implications of transport-induced vibration, which appears to be drone-platform and cargo-specific needs further investigation. Encouragingly, drone-flown insulin has been observed to remain stable when flown by both a fixed-wing and a Vertical Take-Off and Landing (VTOL) drone, but the range of medicines investigated to date is limited [14].

Vibration is an important factor that often affects the quality of medicines, providing the activation energy required to induce conformational changes and degradation [18]. Blood products are susceptible to damage from vibration and shock; notably the break-down of red blood cells, known as ’haemolysis’ [19, 20]. In the case of mAbs, it can cause aggregates to form when protein monomers are subject to various types of vibration as a result of protein exposure to hydrophobic surfaces or air/water interfaces [21].

The mechanism of sub-visible particle formation has been found to vary for different proteins under similar stress conditions [22]. Shake-and-stir induced aggregation is the most common kind of vibration which has been studied during the manufacturing process. The anti-cancer mAb Cituximab has been shown to aggregate when subjected to stirring stress [23]. Such aggregates may also render the biological therapeutics inactive and/or possibly immunogenic. The antigen-binding activity of recombinant single-chain fragment variables or SCFv antibody fragments has been found to reduce with a first-order rate constant of 0.83/h in buffer at shear of 20,000/s [24]. Chemical modifications including oxidation and degradation due to specific consequences or structures have also been commonly observed in stirring or shaking studies [24].

There is growing awareness that evidence should be gathered, as part of routine logistics operations, to demonstrate that the method of transportation will not adversely affect the quality of the medical cargo being transported [11]. Whilst mAbs are therapeutically very potent, they can be structurally quite unstable as being protein-based therapeutics, they are made up of labile molecular sub-sections which can be disrupted when exposed to various physical, thermal, or chemical stressors [23]. The rationale for selecting mAbs was that this class represents the greatest potential for vibrational vulnerability in the physical structure of the molecule. These molecules are also temperature sensitive, with temperature affecting the optimum stability of the product. Where possible (not withstanding low temperature solubility issues) most aseptic products should be stored and transported at 2–8°C to limit potential biological growth, and whilst small temperature excursions above 8°C are acceptable for short periods, freezing of mAbs may result in protein denaturisation and must be avoided [25]. Cytotoxic cancer medicines, for example paclitaxel, are also vulnerable to biological growth and solubility issues.

**Fig 1:**
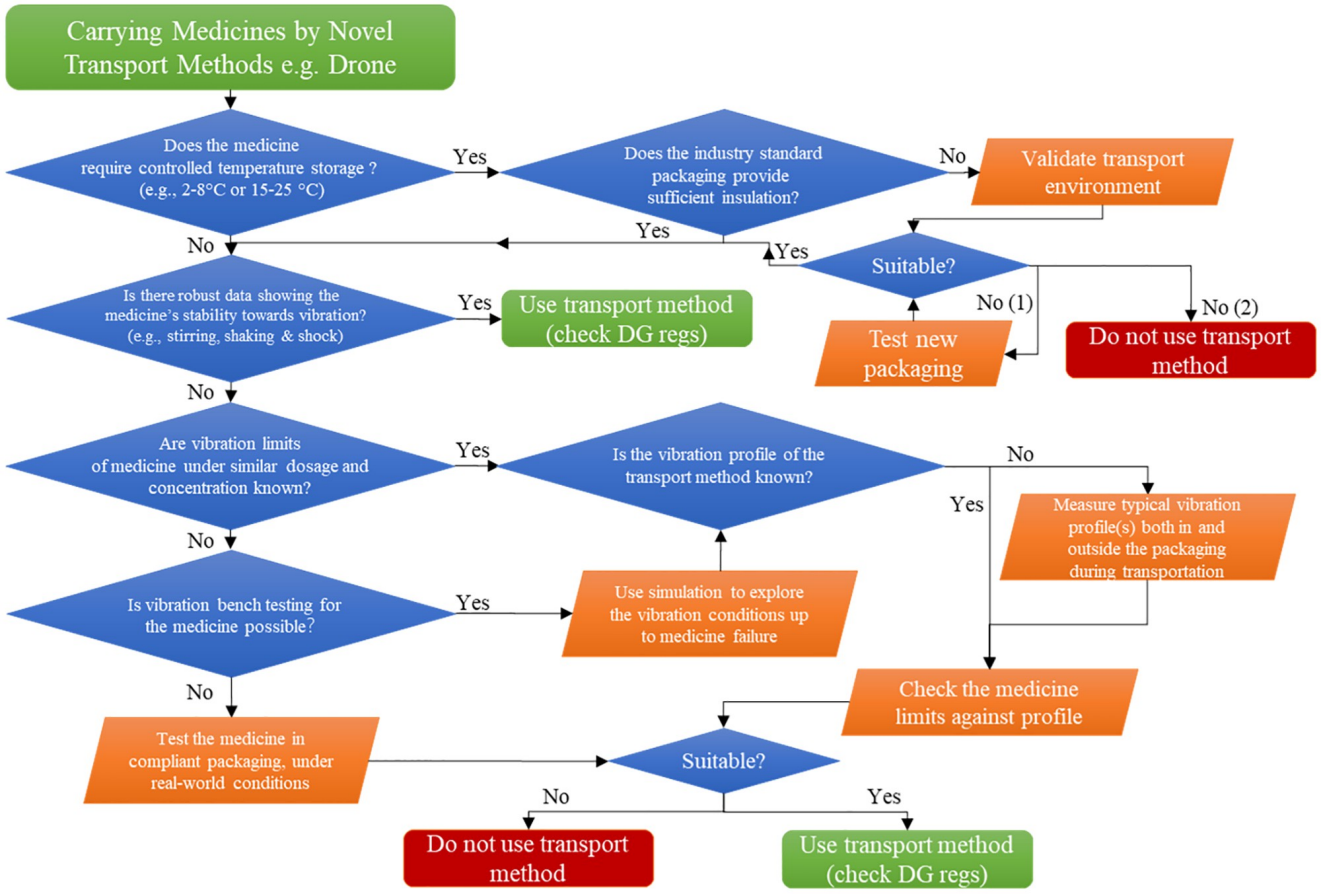
Methodology flow diagram for the validation or novel transport modes to assure quality of medicines, building on UNICEF’s drone delivery decision model [26].

To the best of the authors’ knowledge, such empirical evidence related to the transportation of mAbs by drone does not exist, and this paper reports on the design of practical trials to quantify the potential effects, providing a framework for the quality assurance of flown cancer treatments. When developing novel transport methods for the carriage of medicines and medical products, UNICEF has introduced a delivery decision model, to assess the suitability of the new approach [26]. The authors have applied and expanded this methodology for drones carrying short expiry, unstable cancer medications using current quality assurance practices (Fig 1). The influence of vibration during flight, validated by benchtop experiments, is not currently part of typical testing frameworks and so was a key addition. A condition of the MHRA’s good distribution practice, GDP, is that quality assurance procedures must be applied to the transported medicines, therefore experiments are required that confirm the stability and quality of drone flown monoclonal antibodies, if routine drone deliveries of cancer medicines are to receive regulatory approval.

Thus, the aim of this research was to investigate the influence of drone flight on the stability of three medicines commonly administered to treat cancer. These were monoclonal antibodies, with large biological molecules that require aseptic manufacture before administration and are considered unstable. Investigating drone transportation of such unstable and short expiry medicines provided an opportunity to evaluate UNICEF’s drone delivery decision model and explore the role of vibration within this framework

The core objectives were to:

- Develop a methodology to evaluate and quantify vibration on medical cargoes emanating from the different stages of flight in a VTOL fixed-wing electric drone.
- Quantify if there are negative effects on the safety and quality of oncology treatments due to vibrations emanating from VTOL-Fixed-Wing drone transportation using a real-world trial involving redundant oncology treatments and a Skylift V50, (an adapted version of the Mugin 5 Pro VTOL drone).
- Develop a bench top, in-vitro approach to validate the analytical methodologies by exploring the edge-of-quality failure by applying extremes of rotational vibration to the mAb samples.

## 2. Methodology

The adopted approach builds on a previous study using a fixed-wing combustion engine drone where insulin samples were flown and vibration levels quantified using 3-axis accelerometers [14]. The quality of the flown samples was subsequently tested using the NHS Standard Protocol testing aseptic biopharmaceuticals [25]. Two trials were undertaken as part of this study using a VTOL drone; the first investigated the effects of multiple take-off, hovering, and landing flights; the second investigated the effects of a single flight with take-off, hover, transition, cruise, and landing elements.

### Sample preparation

Three different mAb treatments provided by the IOW NHS Trust were tested in the trials, all being expired (beyond use-by date) but sealed-as-received in infusion bags and could not be used for human treatment. Expired treatments (all IgG1 type antibodies) were used to limit costs and prevent additional demands on manufacturing units during the COVID-19 pandemic. The first trial used samples of Bevacizumab (Avastin, 400 mg in 116 mL 0.9% NaCl, expiry 03/08/2021), whilst the second also used samples of Trastuzumab (Herceptin, 450 mg in 271 mL 0.9% NaCl, expiry 09/08/2021), and Rituximab (Truxima, 1000 mg in 600 mL 0.9% NaCl, expiry 26/09/2021). Although these treatments were no longer suitable for clinical use, they still conformed to the NHS clinical quality criteria relating to colour, clarity, precipitation [25] and were therefore deemed suitable for experimentation.

The treatments originated from St Marys Hospital IOW where they were kept in secure refrigerated conditions. They were transported by rail and road to King’s College London for preparation in the days preceding the experiment, and onwards to the test site using industry standard packaging, maintaining storage temperatures between 2–8°C throughout (S1 and S2 Tables). NHS clinical quality was confirmed by visual inspection immediately prior to flights. They were then exposed to UAV transportation and were subsequently returned to King’s College London for post-flight analysis. Flown samples were transported in an 18 L Versapak [27] with 3x industry standard cool packs (frozen for >24 hours prior to departure). To prevent the samples from freezing, the cool packs were wrapped in bubble-wrap insulation.

**Fig 2:**
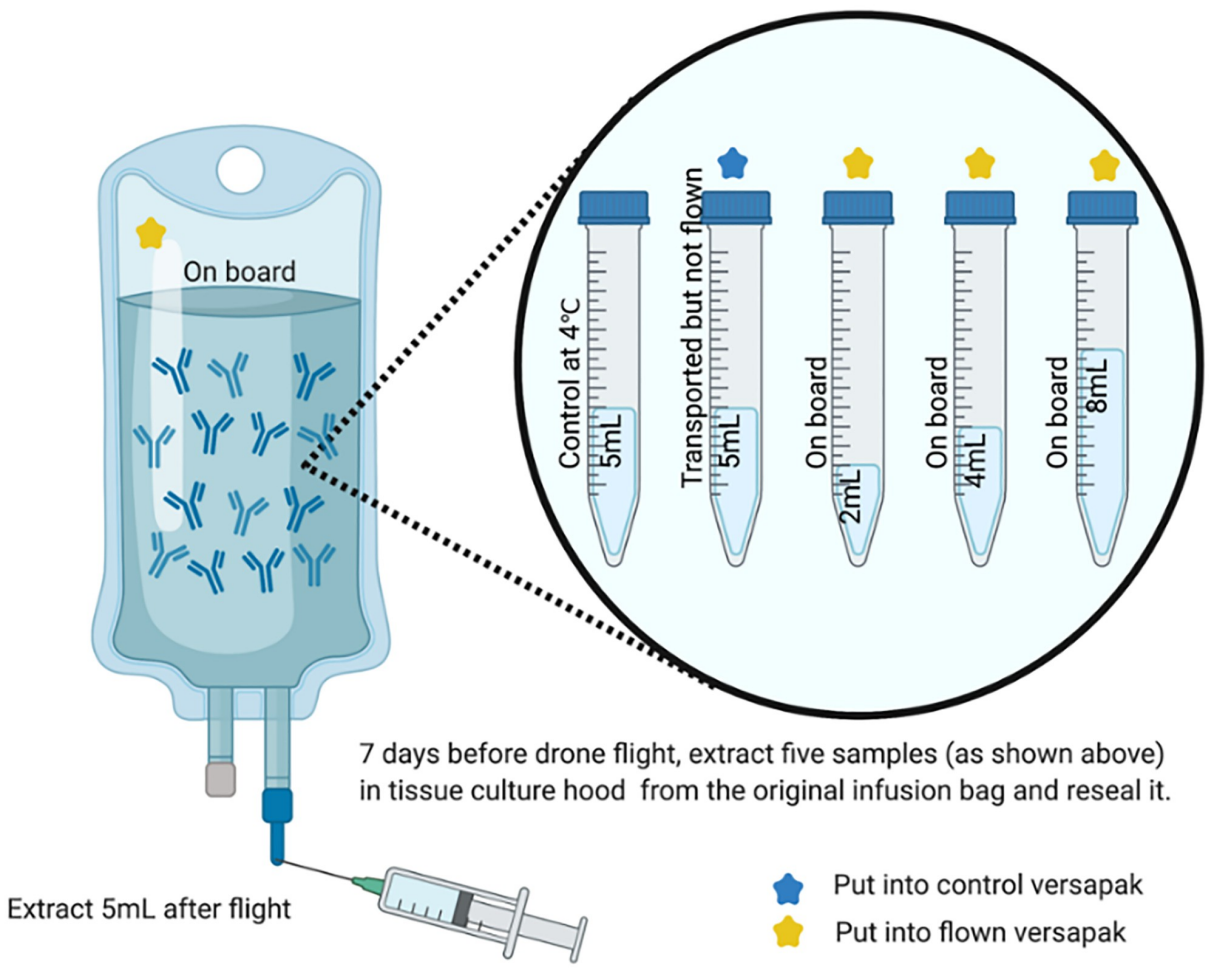
Sample information and preparation process.

The samples themselves were prepared by extracting 24 mL of mAb solution from each sample infusion bag in a controlled tissue culture laboratory environment. These were then deposited in varying amounts into 5x15 mL centrifuge tubes (sterile, greiner bio-one, G188271) after removing the needle (Fig 2, S1 Table) to test the effect of headspace (air gaps) in the infusion bags during transport. The sample tubes that were flown were made of polypropylene with Cat.-No. 188 261 /188 271 / 227 261 / 227 270 which met the pressure requirements for transportation by aircraft. After extraction, the infusion bags (with 80 to 96% of their contents remaining respectively) were also included in the flight tests.

Two control samples were used to isolate the potential effects of factors beyond the drone flight; 1x 5 mL sample which remained in temperature-controlled storage at King’s College London and was not subject to any transportation; and 1x 5 mL sample which was transported in an 18 L Versapak (Trial 1), or a 6 L Versapak (Trial 2) under the same land-based travel conditions as the flown samples but were not flown. A 250 mL saline infusion bag was also transported with the control sample to make the weight of the overall package more akin to that of a full product.

### Instrumentation

**Fig 3:**
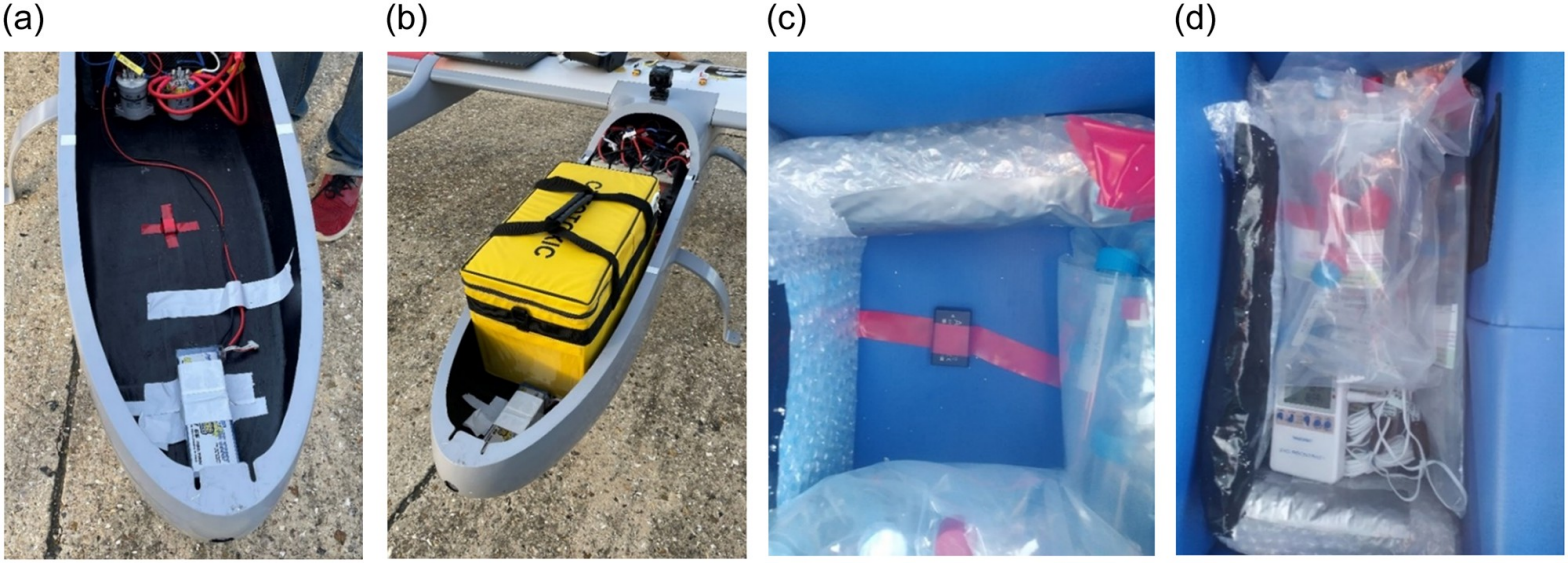
**(a)** Cargo bay with base vibration sensor mounted (held in position by red tape); **(b)** Loaded 18 L Versapak; **(c)** Versapak base sensor; **(d)** Versapak with insulated cool packs, sensors, samples.

To monitor temperatures and vibration levels, a series of sensors accompanied the samples (Table 1, Fig 3). A NIST Certified Traceable^®^ Excursion Trac temperature logger with a glycol bottle probe monitored temperatures in the Versapak containing the flown samples, whilst an RS PRO PRO-USB-1 Temperature Data Logger (Trial 1) or NIST Certified TRACEABLE Sentry Thermometer with a bullet probe (Trial 2) monitored the non-flown samples. The loggers were set to record at 1-min intervals, whilst the thermometer recorded the minimum and maximum values experienced over the trial period (Min. 2°C, Max. 5°C). The temperature monitors indicated that both Versapaks maintained temperatures within the acceptable range (2–8°C) throughout the trials and thus storage specifications were met (S1 Fig).

**Table 1:** Packaging and sensor arrangements for each trial.

Parameter	Trial 1 –Hover Only (28/10/21)			Trial 2 –Cruise (23/11/21)		
	Ctrl 1	Ctrl 2	Flown	Ctrl 1	Ctrl 2	Flown
Medicine/Product Tested	Bevacizumab (exp. 03/08/21)			Bevacizumab (exp. 03/08/21)		
	Saline Solution			Trastuzumab (exp. 09/08/21)		
				Rituximab (exp. 26/09/21)		
Packaging	Storage	VP-M	VP-M	Storage	VP-S	VP-M
Cargo Bay Aclmtr.	-	-	AX3	-	-	AX3
Package Base Aclmtr.	-	-	AX3	-	-	AX3
Thermometer	-	RS-PRO	Bottle	-	Bullet	Bottle
Cooling Packs	Fridge	3x IS	3x IS	Fridge	1x IS	3x IS

VP-M = Medium Versapak, Aclmtr. = Accelerometer, exp. = expired date.

During flights, vibration experienced inside the cargo bay and inside the Versapak was measured using two triaxial MEMS data logging accelerometers (Axivity AX3, axivity.com) set to a sampling rate of 1.6 kHz and a range of ±8 g. The products themselves were not instrumented; instead, the sensor reading on the base of the packaging was assumed to be representative of the vibration exposure subjected to the products.

Placing the accelerometers on the external surface of the infusion bags was not a reliable solution due to the product contents offering a level of isolation. Whilst not possible with the available equipment, the ideal sensor position to monitor the product’s vibration exposure would be within the product itself (i.e., inside the infusion bags).

### Medicine analyses

On return to the laboratory at King’s College London, the stressed samples were analysed for structural integrity by dynamic light scattering (DLS) and size exclusion, high performance liquid chromatography (SE-HPLC), Tables 3 and 4. The approaches used were considered as reference methods in the stability assessment of biopharmaceutical products (ICH Q5C & Q5E) NHS Standard Protocol for Deriving and Assessment of Stability [25].

DLS running on *Malvern ZetaSizer NanoZS90* was used to track the hydrodynamic diameter of protein and aggregates within the instrumental range 0.3 nm to 10000 nm. The peaks in particle size detected were additionally quantified with regards to their polydispersity index (PDI), indicating the breadth in size or conversely, the mono-dispersity of the peaks in sample size (S2 Table). A deviation greater than 1 nm from the hydrodynamic diameter of the representative population of the mAbs was considered to be out of specification. Similarly, the occurrence of another population with an intensity percentage greater than 10% was also considered out of specification. The population was considered monodisperse when the PDI was ≤0.1.

Aggregation (high molecular weight species, HMWS) or fragmentation (low molecular weight species, LMWS) of mAbs [28] was determined by size exclusion chromatography (SEC) using an Agilent 1100 series HPLC system with UV-Vis detector (Agilent AdvanceBio SEC 300Å, 4.6 x 150 mm, 2.7 μm filled with sub–3μm particles (PL1580-3301)). A guard column was attached (Agilent AdvanceBio SEC 300Å, 4.6 x 50 mm, 2.7 μm (PL1580-1301)) and operated within the HPLC equipment (S3 Table). Samples were filtered through a 0.2μm PES filter before testing. According to NHS standard protocols for biopharmaceutical stability [25], the maximum acceptance criteria are a 5% loss in active protein and a maximum 2% relative to the main peak increase in any degradant peaks.

To quantify whether there were any statistically significant differences observed between the flight and control samples, one-way analysis of variance (ANOVA) tests and Student’s t-tests (two-tailed) were undertaken. The results were considered to be significant when the value of p was <0.05 where “***” indicates p<0.001, “**” indicates p<0.01, and “*” indicates p<0.05. (All data are presented as mean ± SD (n = 3)).

**Fig 4:**
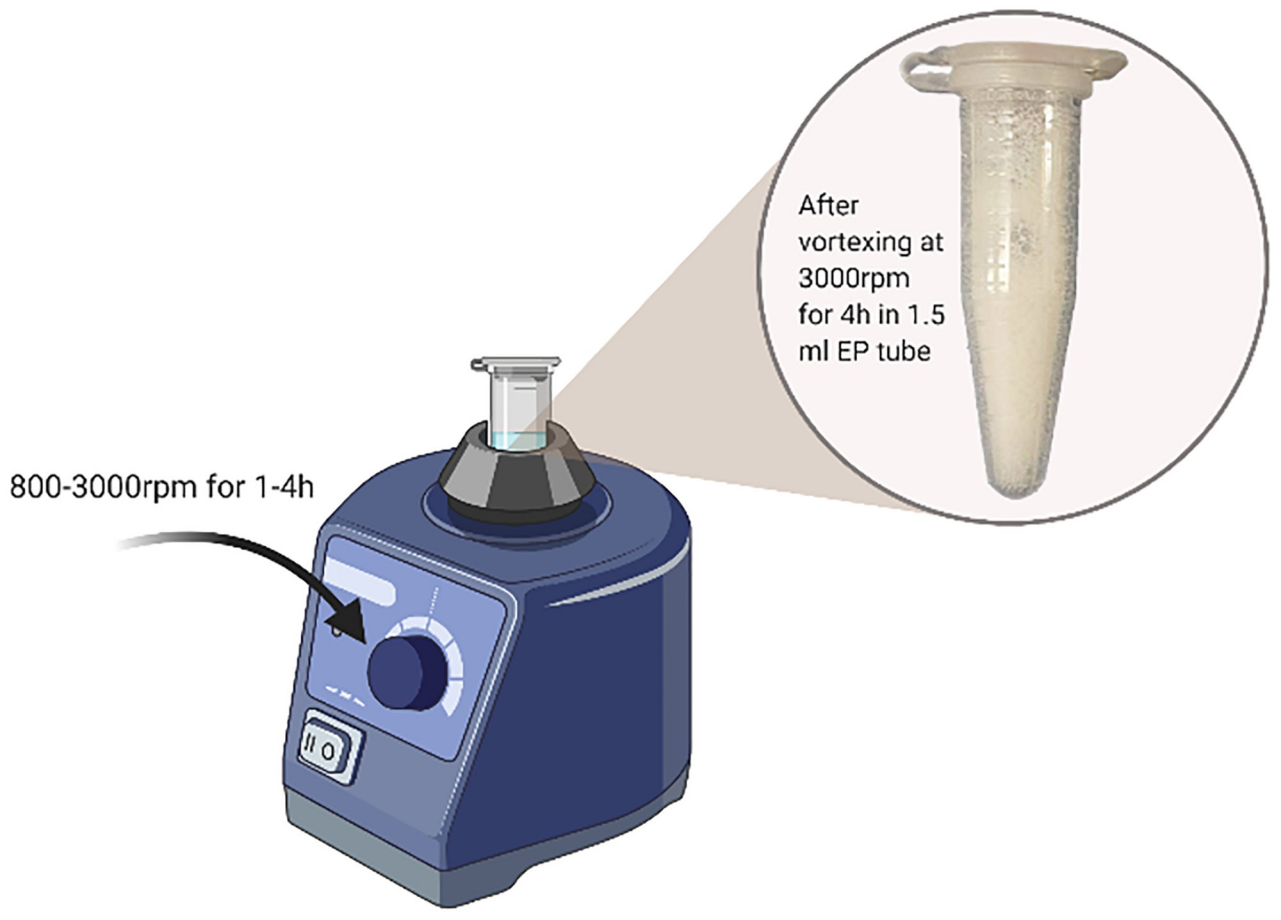
Schematic of forced vortexing experiment used in validating the analysis methods.

Initial vibration simulation was operated on shaker and vortex mixers to create ‘edge-of-failure’ conditions as these machines are available in most laboratories and can apply a range of rotational frequencies over long periods. Thus, they have the capacity to vibrationally stress samples, although such mixers are not a realistic mimic of flight [29]. To validate the detectability of the analytical methods, the three mAbs were also tested for vibration (vortexing) sustainability at different rotation speeds (800-3000rpm). The validation samples were tested in 1.5 mL Eppendorf tubes with negligible headspace (Fig 4).

### Vibration analyses

Vibration analyses were carried out on the data acquired from the triaxial acceleration sensors placed in both the cargo bay and the Versapak. Overall vibration levels were obtained using the standard deviation of the magnitude of the resultant acceleration vector, i.e., the square root of the sum of the variances of the signals in the three orthogonal directions. Octave band spectra were synthesised by first computing the narrowband spectra (not presented here) and summing them in each octave band. Flight data from the drone were temporally synchronised to the triaxial acceleration sensors to enable the various stages of flight to be accurately identified (take-off, hover, transition, cruise, transition, hover, and landing) and the associated implications on vibration assessed.

### Case study platform specifics

**Fig 5:**
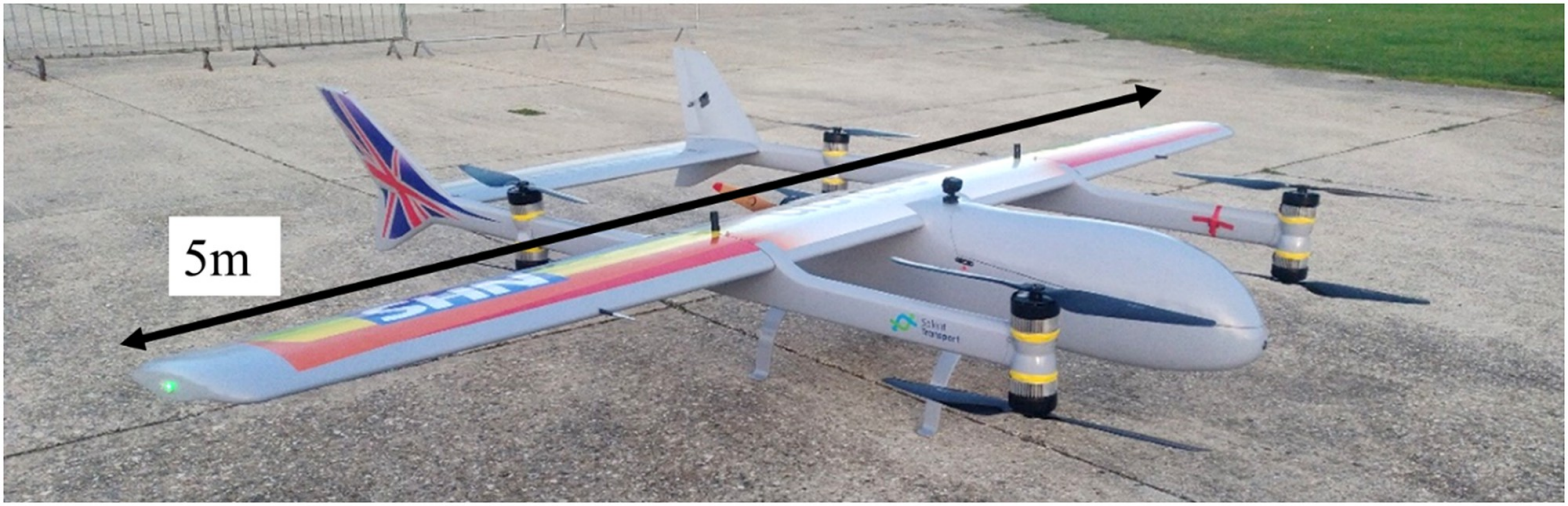
Skylift V50 (Mugin 5 Pro) VTOL drone used in both trials.

The drone tested in the case study was the Skylift V50, an adapted Mugin 5 Pro (https://www.muginuav.com/product/mugin-5-pro-5000mm-vtol-uav-platform-8-motor-mounts/) VTOL-fixed-wing hybrid platform (Fig 5), with a 5 m wingspan and a maximum payload capacity of 20 kg. During the first trial, three flights using only the VTOL motors were undertaken, each of 2 minutes duration and covering the take-off, hover, and landing phases of flight.

**Fig 6:**
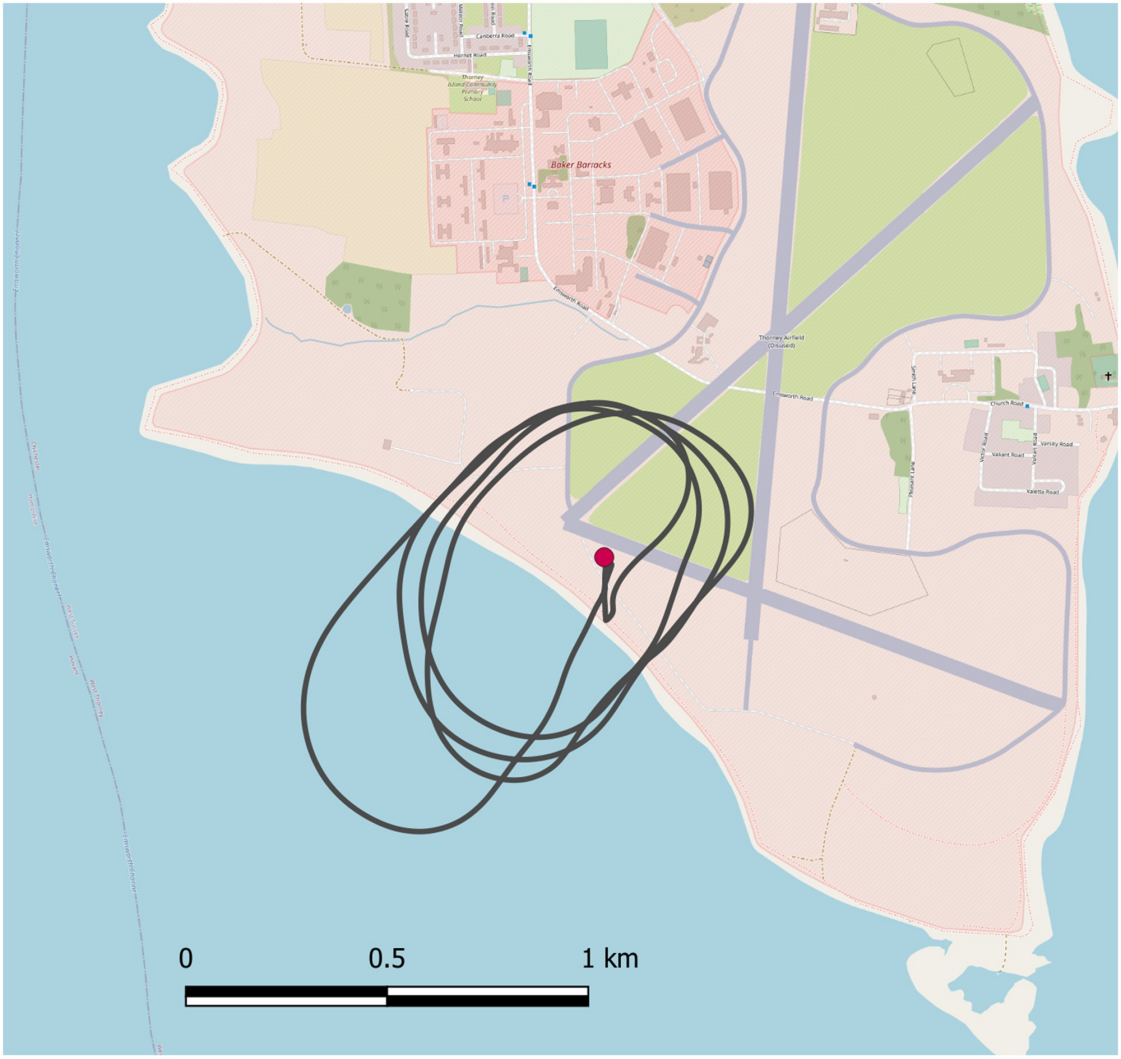
Flight path of the test flight in the second trial, based at the military base at Thorney Island. Distance flown was 10.9km. Base map and data from OpenStreetMap and OpenStreetMap Foundation.

During the second trial, one manually operated flight of 8 minutes was undertaken, during which 6 minutes of fixed-wing cruise flight was achieved, and a distance of 10.9 km was covered (Fig 6). It should be noted that the manual operation may have created greater variability in the vibration profile due to the pilot needing to manually regulate altitude.

## 3. Results

### 3.1 Vibration analysis

**Fig 7:**
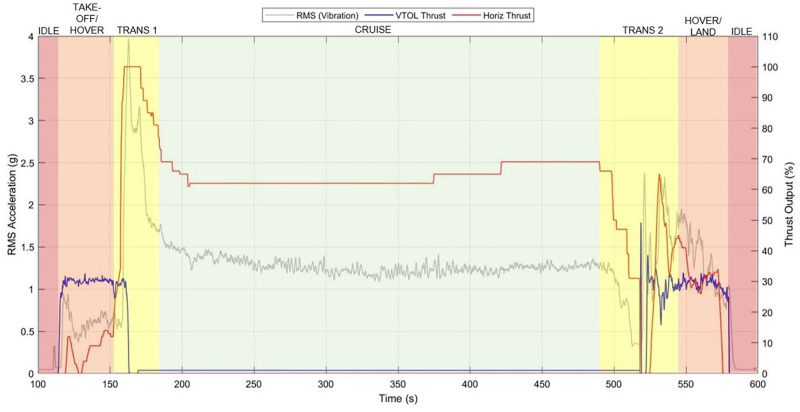
Throttle outputs compared to magnitude of vibration during each stage of flight with the Skylift V50 (Mugin 5 Pro) VTOL drone. High thrust periods correspond with the greatest peaks in vibration measured. Trans 1 = Transition from hover to cruise. Trans 2 = Transition from cruise to hover. The RMS acceleration is calculated with as a 1 second sliding window and the mean is subtracted from the data.

The flight was broken down into the key stages of take-off, hover, transition from vertical to horizontal flight, cruise, transition from horizontal to vertical flight, hover, and landing. Fig 7 demonstrates that the amplitudes of observed vibration were generally greater during periods of transition, which correlated with periods of greatest combined motor thrust. Vibration levels of up to 4 g far exceed those expected in road transportation [14] but generally occur at much higher frequencies where mitigation is easier to achieve. Where the samples were not adversely affected by flight, the vibration findings are supplementary and not a material requirement of this investigation.

**Fig 8:**
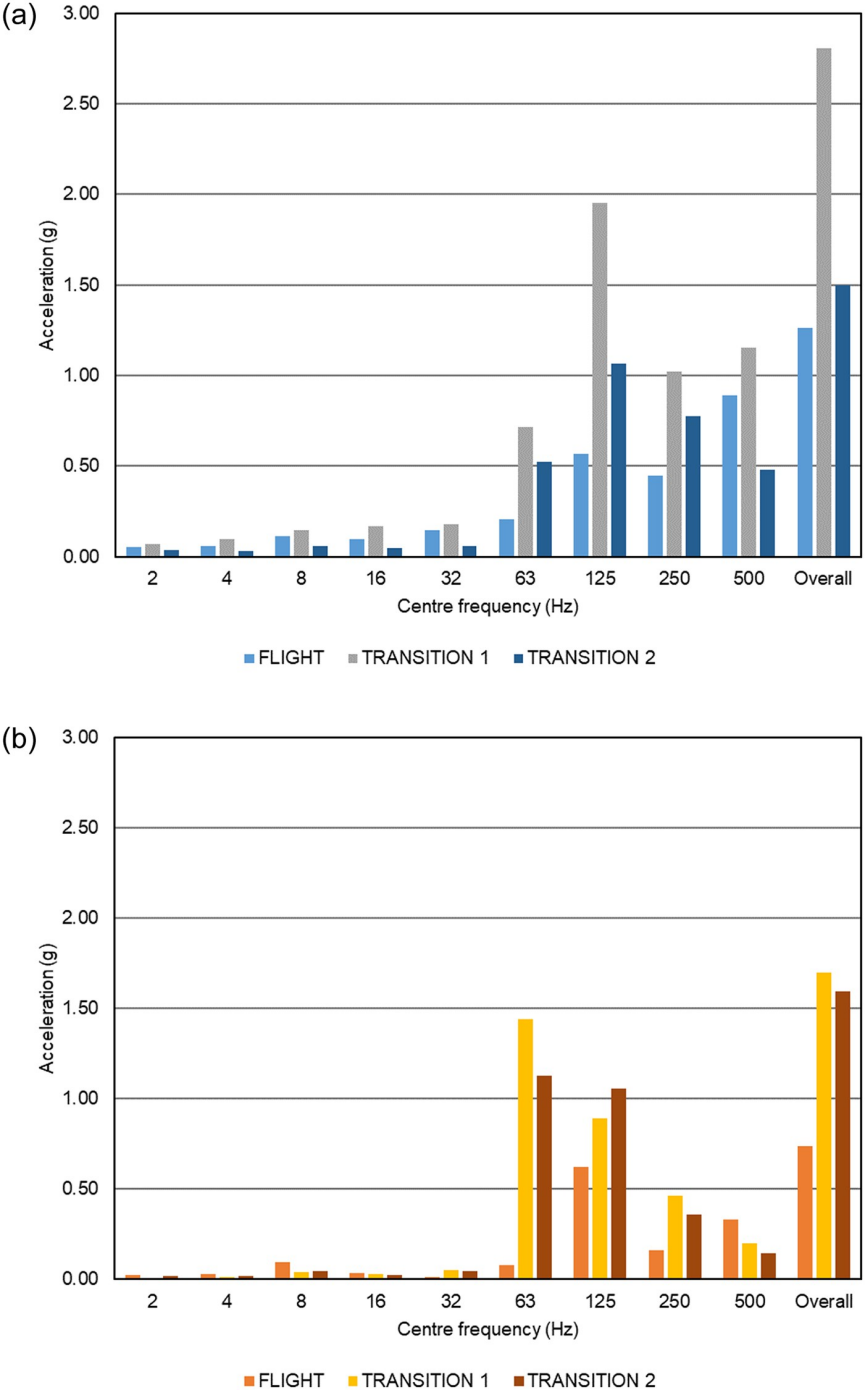
Octave spectra of resultant vibration (a) in the cargo bay of the Skylift V50 (Mugin 5 Pro) and (b) inside the Versapak.

Fig 8 shows octave spectra of the resultant vibration, i.e., accounting for all three translational axes, during the different stages of flight at two measuring positions. Fig 8a shows that vibration in the cargo bay occurs largely above 44 Hz—the lower limit of the 63 Hz octave band—which is consistent with typical rotor speeds during operation and multiples thereof. The vibration was significantly greater in amplitude during transition than forward flight or hover, except at 500 Hz. Fig 8b shows the corresponding spectrum inside the Versapak. In the 63 Hz band, vibration during transition is greater inside the Versapak than in the cargo bay by a factor of 2.0 (transition 1), and 2.1 (transition 2), possibly due to a resonance of the payload within the soft packaging. At higher frequencies, the Versapak was effective at isolating vibration. It should be noted that vibration levels may vary depending on where the sensors are positioned within the cargo bay and Versapak, and between sample tubes. How best to instrument liquid medicines to monitor their vibration exposure is an important issue for future consideration.

### 3.2 Pharmaceutical analysis

**Fig 9:**
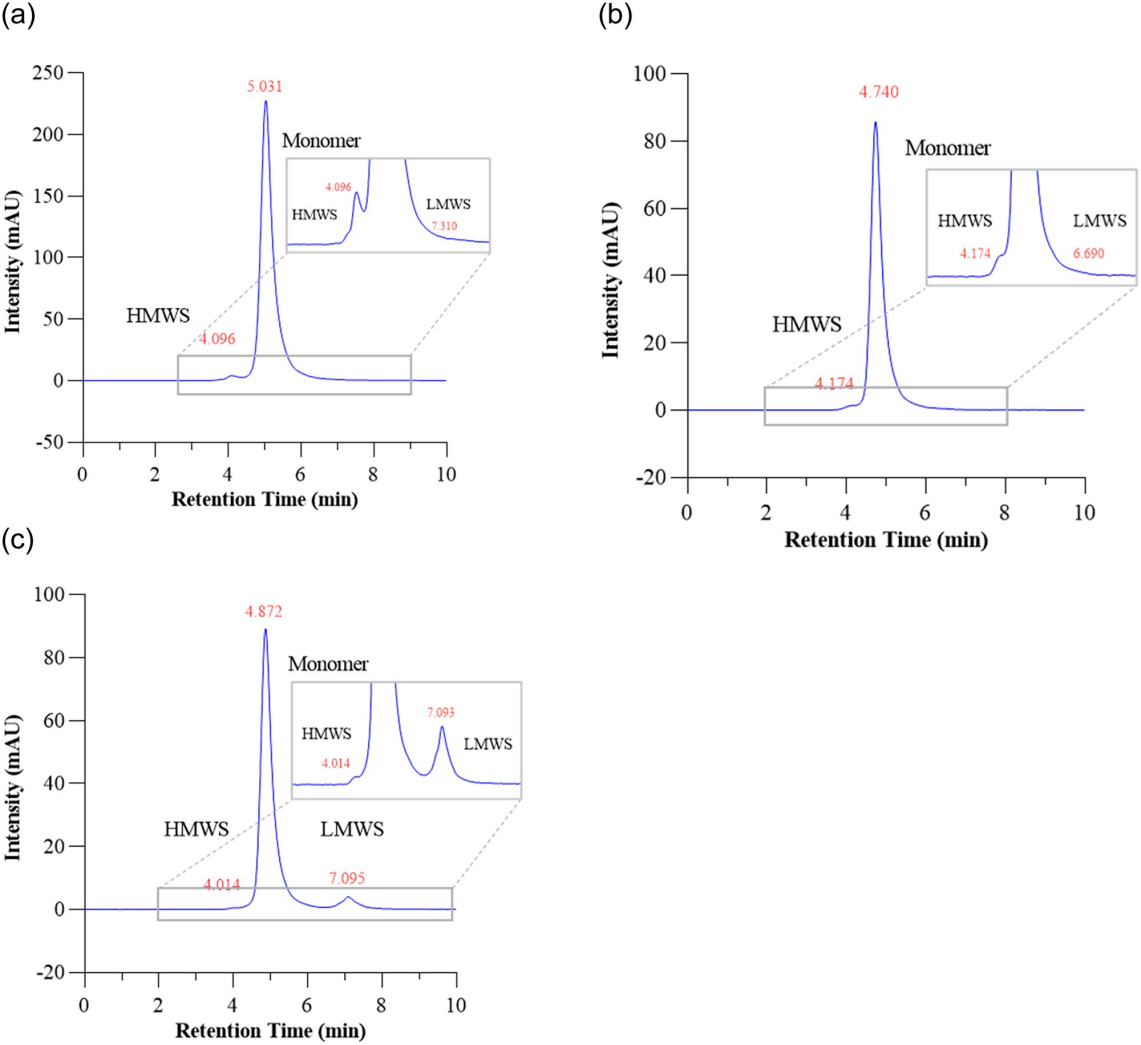
Elution profile of monomeric (a) Bevacizumab, (b) Trastuzumab and (c) Rituximab as received by Size Exclusion Chromatography.

**Fig 10:**
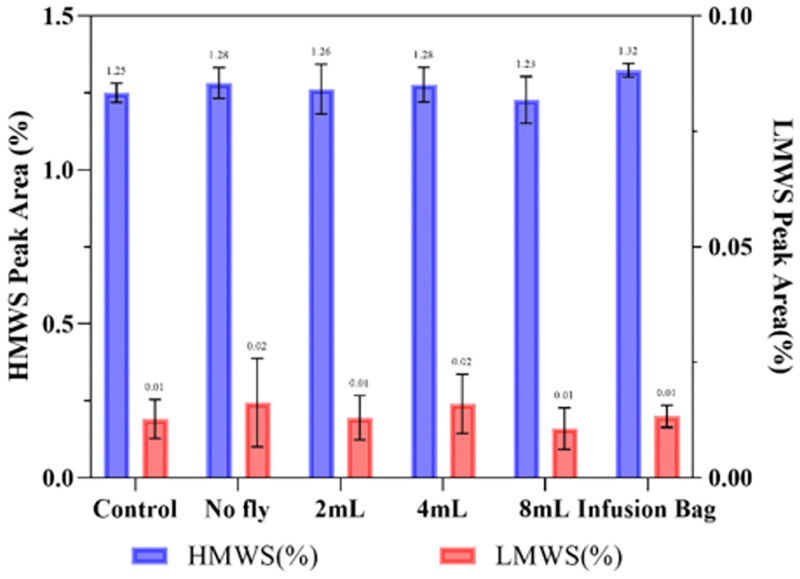
SE-HPLC peak area (%) of Bevacizumab of aggregates (HMWS) and fragments (LMWS) in different storage conditions (trial 2). Data are shown as mean ± SD (n = 3).

The DLS and SE-HPLC methods were validated using mAb samples exposed to different vortex conditions to encourage aggregation and fragmentation, following similar approaches used to simulate the extremes of transport conditions [21]. In these scenarios, aggregation or fragmentation was detected, but each mAb showed a different susceptibility towards vortexing (rotationally-induced vibration, Figs 9 & 10). This corroborates previous studies, where Gokhale et al. [30] shook 25 mg/mL pH = 6.2 Bevacizumab solution at 1000rpm and the aggregates % increased from 1.6% (0h) to 1.9% (24h) and 2.0% (48h). Strømme [31] applied a cap mixer at 4300rpm and within 90 seconds, sufficient visible aggregates could be produced. It should be noted that the concentrations of the flown mAbs were considerably less than those reported in the literature that have studied the effects of vibration, as the samples transported by drone had been pre-diluted ready for administration to the patient, making aggregation in the product less likely. However, Giannos et al. [32] found that when Bevacizumab (Avastin) was diluted ready for administration with 0.9% w/v NaCl, there was a 40–50% loss of monomer concentration and this loss of drug binding activity at low concentrations was due to reversible aggregation such as antibody-surface adsorption, antibody-antibody coupling and a combination of both. Such opposing observations indicate the need for continued vigilance and development with regards to mAb quality control practices.

Pabari et al. [33] found that shaking trastuzumab (0.4 mg/mL) resulted in a 2-fold increase in the average particle size; however, a much higher concentration (22 mg/mL) was resistant to vortexing for 10 minutes at 3600 rpm, but after 60 minutes aggregation (16.2%) was observed. The initial resistance could be associated with the presence of polysorbate 20 in the formulation, accumulated at the air-liquid interface leading to the protection of proteins against interface-related stress hence decreasing the risk of aggregate formation due to agitation [34]. Such work illustrates the complex nature of mAbs sensitivity towards vibration, and the need to validate over a range of vibration/vortexing speeds. Strong evidence of the vibration stability of trastuzumab has been provided by the U.S. Food and Drug Administration (FDA) [35]. A test of Trazimera (trastuzumab) derived from an agitation stress was conducted to evaluate the effect of mechanical/shear stress on product quality during transportation. The worst-case conditions expected during transportation were examined (3000 rpm vortex for 4h at 25°C), resulting in deamidation levels of HC Asn55 (Heavy Chain, asparagine at position 55), suggesting that a vortex/agitation stress does not appear to trigger increased deamidation, indirectly suggesting that transportation is not likely to affect deamination level [35].

Ahmadi et al. [36] found that rituximab samples that were stir-stressed for 30 mins, 200 rpm (1 mg/mL, pH = 7.4) were shown to achieve low levels of sub-visible aggregates (<3%), which enhanced dendritic cells maturation and antigen presentation (CD4^+^ T cells) using fluorescent microscopy [36]. Extended periods of stirring, for example up to 120 hours at 600 rpm, will eventually induce significant aggregation. In the case of 1 mg/mL Rituximab, aggregates within the 0.1 to 1 μm range were observed [37]. The internal filter, present when infusion bags are administered, would not be able to remove all of the aggregates as the filter’s cut-off is 0.22 μm. However, such extended periods of agitation and stress are not likely to be encountered in regular drone delivery operations.

The HPLC results showed that the HMWS (aggregates), mAbs monomer, and LMWS (fragments) were separated from the main chromatographic peak in less than 9 minutes for all three mAbs. The HMWS% values were comparable with those already reported in the literature [38] but varied with equipment, mobile phase (content of phosphate buffer and salt), sample injection volume, and most importantly, preparation time (Fig 9). The small amount of HMWS and LMWS was produced initially during manufacturing or formed naturally, and the relative difference in % with the experimental group was applied to decide the quality of stressed mAbs. All the flown mAb solutions were found to be clear and colourless with no visible particulate material present, irrespective of concentration. The forced vortexing samples produced different degrees of bubbles which disappeared after resting overnight, e.g., the experimental group at 3000 rpm for 4h (Fig 4). Bench tests using a vortex mixer suggested the three mAbs had different sensitivities towards vibration and a higher rotation speed did not correlate with more aggregates (data is shown in S4 Table and S2 Fig). Compared to SE-HPLC, which was used to detect soluble aggregates or degradants sub 0.2 μm, the DLS results suggested that the size changes of the whole protein solution were more distinct (S4 Table, S2 Fig).

### Bevacizumab

The vibration induced by the drone during all phases of the flight was not found to have any detrimental effect on the structural integrity of Bevacizumab, as determined by DLS and SE-HPLC (Table 2 & Fig 10). No aggregation or degradation was observed when the Bevacizumab solution of ~4.8 mg/mL experienced drone flight, with the particle size remaining as one peak at 11.7±0.1 nm with PDI<0.1 and HMWS 1.26±0.04%, LMWS 0.01±0.02%. Comparing the HMWS% to the non-transported control sample, the flown sample had a p = 0.11 (>0.05), which indicated that there was no significant difference after 8 mins of flight in the drone. There was also no change in aggregation and degradation levels in terms of headspace. These results suggest that Bevacizumab [39], (formulated with Trehalose dihydrate and Polysorbate 20) was tolerant to the short drone flight and the associated vibrations in this particular instance.

**Table 2:** Summary of DLS result of Bevacizumab at different storage conditions (trial 2).

Sample	Size (nm)	PDI	Stability
Controlled at 4°C	11.8±0.17	0.057±0.001	Not Applicable
Transported but not Flown	11.8±0.17	0.080±0.015	✓
Flown: 2 mL in 15 mL tube	11.6±0.14	0.044±0.004	✓
Flown: 4 mL in 15 mL tube	11.6±0.11	0.062±0.017	✓
Flown: 8 mL in 15 mL tube	11.6±0.07	0.059±0.004	✓
Flown: Infusion Bag	11.6±0.05	0.056±0.012	✓

All values were expressed as the mean of 3 tests (128 runs) ± Standard deviation. The stability is expressed as: ✓ stable (quality maintained), × not stable.

### Trastuzumab

**Fig 11:**
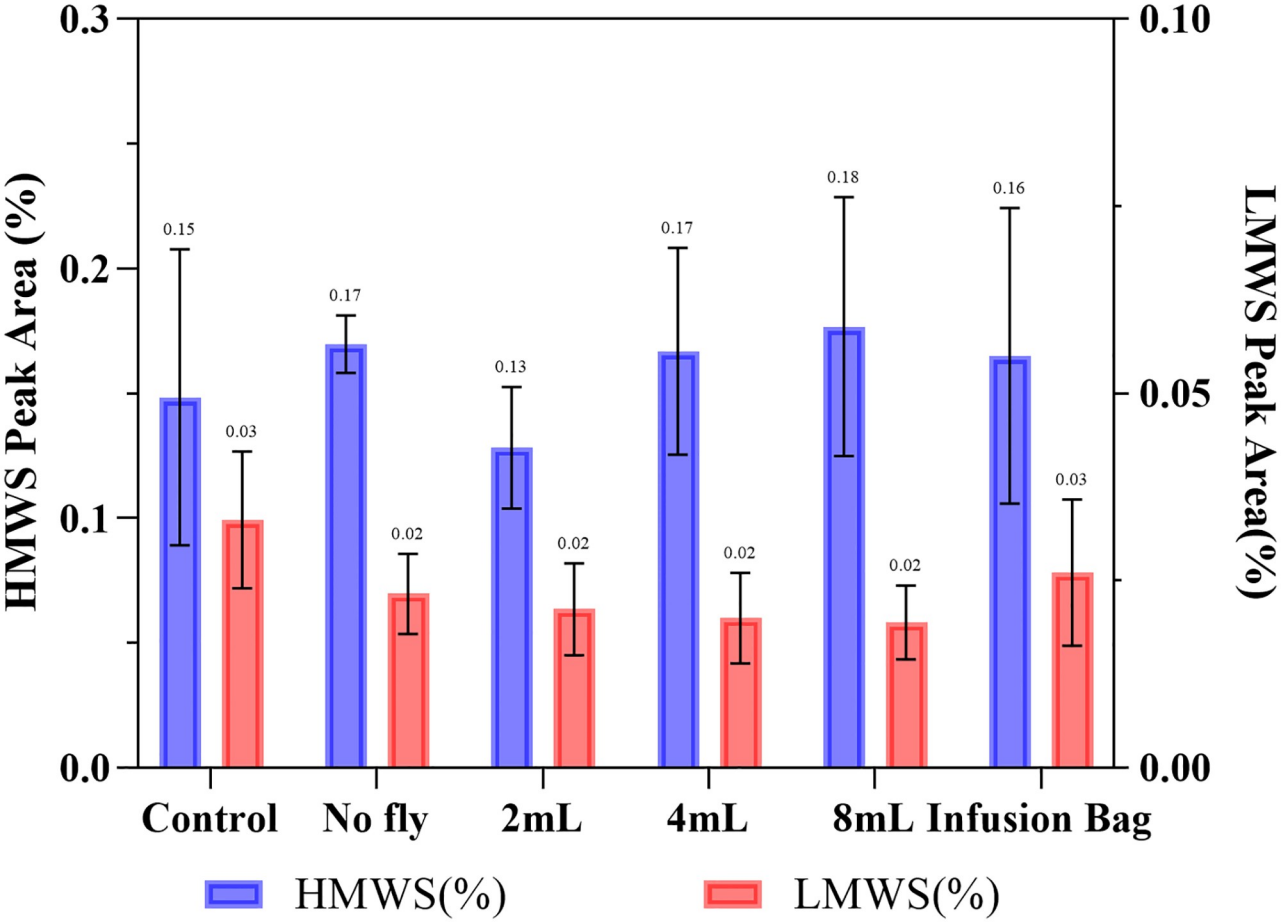
SE-HPLC peak area (%) of Trastuzumab of aggregates (HMWS) and fragments (LMWS) in different storage conditions. Data are shown as mean ± SD (n = 3).

The vibration was also not found to have any detrimental effect on the structural integrity of Trastuzumab, as determined by DLS and SE-HPLC (Table 3 & Fig 11) with no aggregation or degradation being observed when a solution of ~1.7 mg/mL experienced flight by drone, with the particle size remaining in one peak at 11.3±0.1 nm with PDI<0.1 and HMWS 0.15±0.03%, LMWS 0.02±0.01%. Comparing the HMWS% to the non-transported control sample, the flown sample had a p = 0.73 (>0.05), which indicates there was no significant difference after 8 minutes of continuous flight in the drone.

**Table 3:** Summary of DLS result of Trastuzumab at different storage conditions.

Sample	Size (nm)	PDI	Stability
Controlled at 4°C	11.2±0.05	0.067±0.012	Not Applicable
Transported but not Flown	11.2±0.08	0.051±0.017	✓
Flown: 2 mL in 15 mL tube	11.4±0.07	0.062±0.022	✓
Flown: 4 mL in 15 mL tube	11.4±0.14	0.083±0.015	✓
Flown: 8 mL in 15 mL tube	11.2±0.16	0.085±0.007	✓
Flown: Infusion Bag	11.3±0.13	0.071±0.012	✓

All values were expressed as the mean of 3 tests (128 runs) ± Standard deviation. The stability is expressed as: ✓stable (quality maintained), × not stable.

### Rituximab

**Fig 12:**
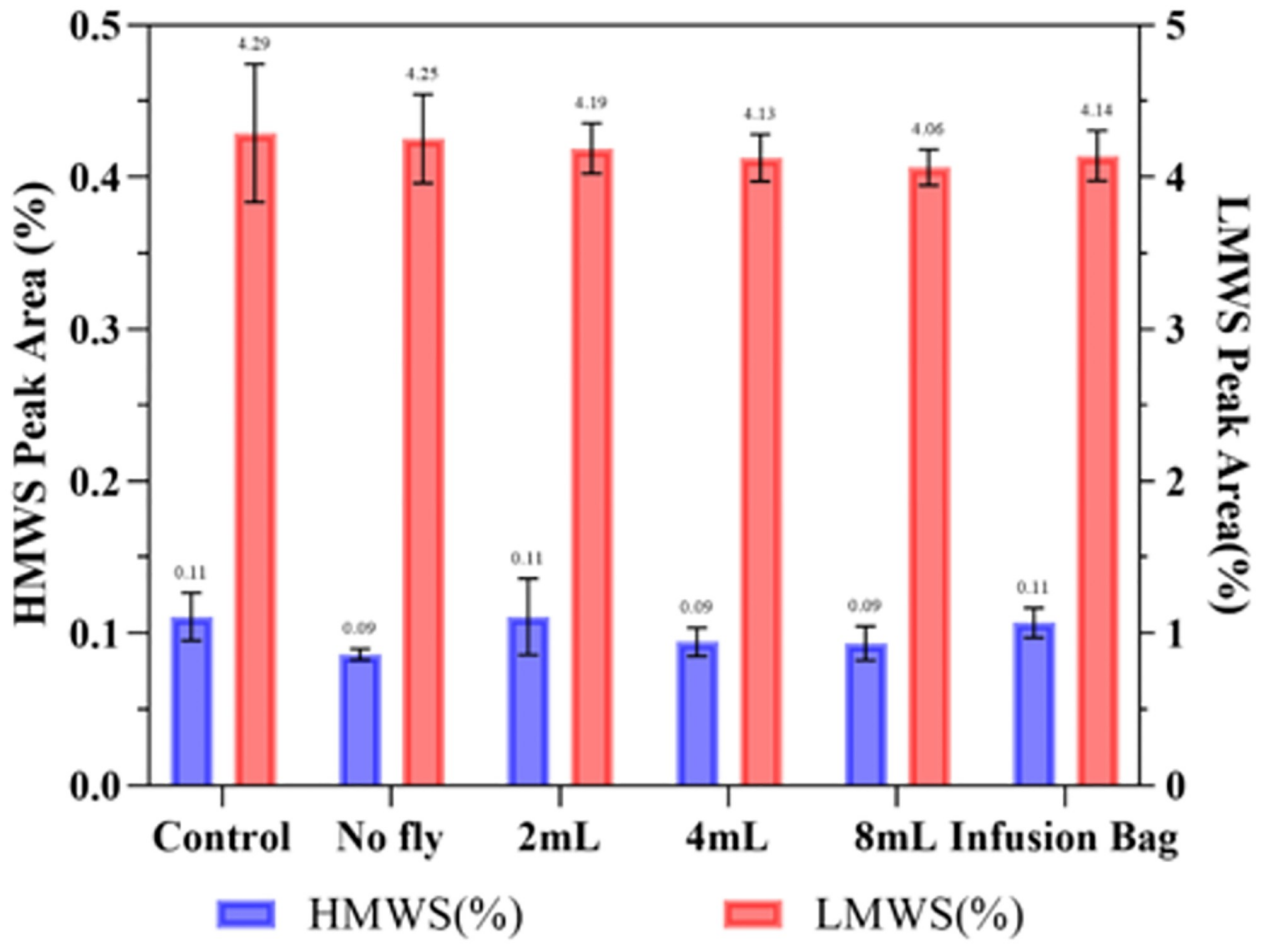
SE-HPLC peak area (%) of Rituximab of aggregates (HMWS) and fragments (LMWS) in different storage conditions. Data are shown as mean ± SD (n = 3). Data are shown as mean ± SD (n = 3).

Similar to the other medicines, the findings suggested that vibration from the drone did not have any detrimental effect on the structural integrity of Rituximab as determined by DLS and SE-HPLC (Table 4 & Fig 12) with no aggregation or degradation being observed when Rituximab solution of ~1.7 mg/mL experienced drone flight, with the particle size remaining in one peak at 11.6±0.3 nm with PDI<0.1 and HMWS 0.10±0.02%, LMWS 4.18±0.11%. Comparing the HMWS% of the flown sample to the control sample suggested that p = 0.75 (>0.05), indicating no significant difference after 8 minutes of flight.

**Table 4:** Summary of DLS result of Rituximab at different storage conditions.

Sample	Size (nm)	PDI	Stability
Controlled at 4°C	11.4±0.27	0.058±0.011	Not Applicable
Transported but not Flown	11.6±0.20	0.085±0.009	✓
Flown: 2 mL in 15 mL tube	11.5±0.30	0.077±0.024	✓
Flown: 4 mL in 15 mL tube	11.8±0.11	0.072±0.025	✓
Flown: 8 mL in 15 mL tube	11.9±0.13	0.057±0.022	✓
Flown: Infusion Bag	11.5±0.05	0.082±0.012	✓

All values were expressed as the mean of 3 tests (128 runs) ± Standard deviation. The stability is expressed as: ✓stable (quality maintained), × not stable.

## 4. Discussion

The favourable stability results are consistent with the protective effect of the surfactants present in the formulation and the use of appropriate infusion bags formed from low-adherence polyolefin/polyamide (PL-2442) [40]. Furthermore, mAb infusions are connected to a sterile, non-pyrogenic, low protein binding filter (0.2um to 1.2um) for patient safety, thus mAbs aggregation will lower the available dose whereas fragments may pass through, potentially endangering patients [41]. We report the stability and quality of drone-flown mAbs with respect to the quaternary structure using two analytical approaches frequently applied in mAbs quality assurance [25]. Maintenance of the quaternary structure directly relates to mAb activity in-vivo and thus the quality of the medicine. Rotational vibration applied using a vortex mixer validated the analysis, as aggregation and fragmentation were detected at specific vortex rpm settings (Fig 10). This methodology provided a convenient in-vitro test to explore the edge of mAb failure in relation to vibration.

The permanent unfolding of the protein secondary structure was assumed to be negligible; Fourier transform infrared spectroscopy (FT-IR) investigations of the three mAbs supported this hypothesis, with no statistical differences between the spectra for the control and mAbs exposed to vibration. Thus, aggregation and fragmentation were the focus of the investigation reported here.

The present study has shown that Bevacizumab (*Avastin*), Trastuzumab (*Herceptin*), and Rituximab (*Truxima*), are not affected by transportation in the V50 VTOL drone during vertical ascent, decent, transition and fixed-wing horizontal flight of up to 8-minutes. The effect of interfaces, which are divided into the air-liquid interface, as a consequence of headspace; and the container-liquid interface, are not significant at this level of vibration. Moreover, no significant reduction was found in the main peak area of the monomer during the SE-HPLC experiment, which further indicated that the drug quality of the three mAbs had remained. These findings are encouraging in relation to the future transportation of aseptically prepared mAbs medicines used for cancer treatment by drone.

When adjustments are made to transportation modes, good distribution practice requires experimental evidence showing medicine quality, and thus safety, have been maintained [11]. Drones, being a novel transport method, may have unknown effects on medicines, necessitating careful monitoring and evaluation. The work reported here has contributed to our understanding of the structural stability of mAbs when exposed to drone flight by showing no change in aggregation and fragmentation within three different mAbs prepared for use in the clinic. Nevertheless, as mAbs’ functional properties (bioactivities) are critical, the authors’ plan to investigate the effects of vibration in future studies. This need is driven by the wide range of transportation vehicles available, the paucity of research into the effects of vibration on sensitive medications and the role of additives. For example, aggregation within cetuximab infusions may be reduced from 25% to 2% with the introduction of polysorbate and glycine [42]. In such work, the adsorption of IgG1 at the air-water interface was found to induce aggregation, which can be minimized by the addition of surfactants, such as polysorbate 20 and polysorbate 80 [43]. Additionally, because of their sensitivity towards vibration and the consequences of rapid expulsion from a syringe, the reconstitution of mAbs is often recommended by manufacturers to be by gentle swirling and not shaking, such insights need to be taken into account [41].

### Limitations/future research

The present study used expired medicines that were redundant for human use to minimise costs and prevent additional demands on the manufacturing unit during the COVID-19 pandemic. Future research would seek to use in-date/non-expired products to validate the findings of this work, in addition to identifying the limits of the medicines on a product-by-product basis. Eventually, a set of known vibration and shock limits (frequency content, amplitude, duration, direction), for different medicines and other medical products (e.g., blood units) can be determined through controlled vibration bench testing. This will eliminate the need to use live products when validating transport methods, as measured vibration can be checked against a set of lookup tables, specific to each product.

Aside from the vibration issues, it may also be beneficial for future work to demonstrate that low temperature minima, which may induce precipitation in aseptic medicines, are not exceeded as a result of sustained drone flights at different outside temperatures.

This study investigated the effects of short VTOL flights with a cruise element. It is expected that the duration of regular operations, for example, flights between the mainland and St. Mary’s Hospital would be 30+ minutes. It is currently unknown how duration affects the cargoes; thus, confirmation of their stability over sustained flight would be recommended. The findings of this paper, the improvements in quality assurance and the framework developed therein, will facilitate these investigations into sustained flight.

## Supporting information

S1 Table Sample configurations during trials. IV = Intravenous (Infusion). Infusion bags were not full due to extractions for the other containers, e.g., 100-5-5-2-4-8 = 76 mL.
(DOCX)

S2 Table DLS analysis parameters. (DOCX)

S3 Table SE-HPLC analysis parameters. (DOCX)

S4 Table Summary of DLS result of Bevacizumab, Trastuzumab and Rituximab at different vibration (vortex) conditions. All values were expressed as the mean of 3 tests (128 runs) ± Standard deviation. The stability is expressed as: ✓stable (quality maintained), × not stable.
(DOCX)

S1 Fig Temperature monitoring during the experiment. The decrease in temperature after 10:30 occurred after loading.
(DOCX)

S2 Fig (a) Bevacizumab, (b) Trastuzumab, (c) Rituximab vibration (vortex) structural integrity determined by SE-HPLC. Data are shown as amounts of HMWS or LMWS (expressed in% of the main peak) ± SD (n = 3).
(DOCX)
