# Supplementary material for: Investigating the influence of drone flight on the stability of cancer medicines
Source: PLoS One. 2023 Jan 6;18(1):e0278873. doi: 10.1371/journal.pone.0278873 (PMC9821719; doi:10.1371/journal.pone.0278873)
Supplement: S1 Table — IV = Intravenous (Infusion). Infusion bags were not full due to extractions for the other containers, e.g., 100-5-5-2-4-8 = 76 mL. (DOCX) [file pone.0278873.s001.docx]

***S1 Table.*** *Sample configurations during trials. IV = Intravenous (Infusion). Infusion bags were not full due to extractions for the other containers, e.g., 100-5-5-2-4-8 = 76 mL.*

| Medicine | Trial 1 – Hover Only (28/10/21) | | | Trial 2 – Cruise (23/11/21) | | |
| --- | --- | --- | --- | --- | --- | --- |
|  | Ctrl 1 | Ctrl 2 | Flown | Ctrl 1 | Ctrl 2 | Flown |
| Bevacizumab | 5 mL in 15 mL Tube | 5 mL in 15 mL Tube | 76 mL 100 mL IV Bag  2 mL in 15 mL Tube  4 mL in 15 mL Tube  8 mL in 15 mL Tube | 5 mL in 15 mL Tube | 5 mL in 15 mL Tube | 76 mL in 100 mL IV Bag  2 mL in 15 mL Tube  4 mL in 15 mL Tube  8 mL in 15 mL Tube |
| Trastuzumab | - | - | - | 5 mL in 15 mL Tube | 5 mL in 15 mL Tube | 226 mL in 250 mL IV Bag  2 mL in 15 mL Tube  4 mL in 15 mL Tube  8 mL in 15 mL Tube |
| Rituximab | - | - | - | 5 mL in 15 mL Tube | 5 mL in 15 mL Tube | 476 mL 500 mL IV Bag  2 mL in 15 mL Tube  4 mL in 15 mL Tube  8 mL in 15 mL Tube |
| Saline Solution | - | 250 mL IV Bag | - | - | 250 mL IV Bag | - |
