# Supplementary material for: Investigating the influence of drone flight on the stability of cancer medicines
Source: PLoS One. 2023 Jan 6;18(1):e0278873. doi: 10.1371/journal.pone.0278873 (PMC9821719; doi:10.1371/journal.pone.0278873)
Supplement: S2 Table — (DOCX) [file pone.0278873.s002.docx]

***S2 Table.*** *DLS analysis parameters*

| Material Refractive Index | 1.450 |
| --- | --- |
| Dispersant | Water |
| Temperature | 25℃ (equilibrate 120 seconds) |
| Measurement Angle | 173° Backscatter |
| Sample Volume | ~0.7mL (1.0-1.5cm height) |
| Cell | DTS0012 disposable Cuvettes |
