# Supplementary material for: Investigating the influence of drone flight on the stability of cancer medicines
Source: PLoS One. 2023 Jan 6;18(1):e0278873. doi: 10.1371/journal.pone.0278873 (PMC9821719; doi:10.1371/journal.pone.0278873)
Supplement: S3 Table — (DOCX) [file pone.0278873.s003.docx]

***S3 Table.*** *SE-HPLC analysis parameters*

| Mobile Phase | 0.1mM Potassium phosphate buffer + 0.25M KCl pH=7.0 |
| --- | --- |
| Flow rate | 0.35 mL/min |
| Temperature | 25℃ |
| Injection Volume | 5μL |
| Detection Wavelength | 280nm |
| Acquisition Time | 15 mins |
