# Supplementary material for: Investigating the influence of drone flight on the stability of cancer medicines
Source: PLoS One. 2023 Jan 6;18(1):e0278873. doi: 10.1371/journal.pone.0278873 (PMC9821719; doi:10.1371/journal.pone.0278873)
Supplement: S4 Table — All values were expressed as the mean of 3 tests (128 runs) ± Standard deviation. The stability is expressed as: ✓stable (quality maintained), × not stable. (DOCX) [file pone.0278873.s004.docx]

**S5 Table.** Summary of DLS result of Bevacizumab, Trastuzumab and Rituximab at different vibration (vortex) conditions. All values were expressed as the mean of 3 tests (128 runs) ± Standard deviation. The stability is expressed as: 🗸stable (quality maintained), × not stable.

| Active ingredient | Sample | Size (nm) | PDI | Stability |
| --- | --- | --- | --- | --- |
| Bevacizumab | Controlled at 4°C | 11.9±0.57 | 0.057±0.023 | Not Applicable |
|  | 800rpm for 1h | 11.8±0.17 | 0.062±0.018 | 🗸 |
|  | 1200rpm for 1h | 21.1±0.25 | 0.040±0.018 | × |
|  | 1600rpm for 1h | 12.0±0.36 | 0.015±0.014 | 🗸 |
|  | 2400rpm for 1h | 11.7±0.53 | 0.088±0.004 | 🗸 |
|  | 3000rpm for 4h | 11.9±0.37 | 0.093±0.003 | 🗸 |
| Trastuzumab | Controlled at 4°C | 11.6±0.10 | 0.066±0.023 | Not Applicable |
|  | 800rpm for 1h | 20.9±0.18 | 0.092±0.021 | × |
|  | 1200rpm for 1h | 15.7±0.51 | 0.021±0.008 | × |
|  | 1600rpm for 1h | 12.6±0.62 | 0.082±0.008 | 🗸 |
|  | 2400rpm for 1h | 13.2±0.60 | 0.041±0.027 | × |
|  | 3000rpm for 4h | 12.2±0.17 | 0.071±0.025 | 🗸 |
| Rituximab | Controlled at 4°C | 11.2±0.32 | 0.022±0.012 | Not Applicable |
|  | 800rpm for 1h | 11.7±0.34 | 0.034±0.011 | 🗸 |
|  | 1200rpm for 1h | 11.8±0.14 | 0.028±0.012 | 🗸 |
|  | 1600rpm for 1h | 11.8±0.11 | 0.072±0.025 | 🗸 |
|  | 2400rpm for 1h | 19.4±4.71 | 0.140±0.021 | × second peak at 541.1nm |
|  | 3000rpm for 4h | 15.5±1.52 | 0.290±0.019 | × second peak at 613.6nm |
