# Supplementary material for: Investigating the influence of drone flight on the stability of cancer medicines
Source: PLoS One. 2023 Jan 6;18(1):e0278873. doi: 10.1371/journal.pone.0278873 (PMC9821719; doi:10.1371/journal.pone.0278873)
Supplement: S1 Fig — The decrease in temperature after 10:30 occurred after loading. (DOCX) [file pone.0278873.s005.docx]

*
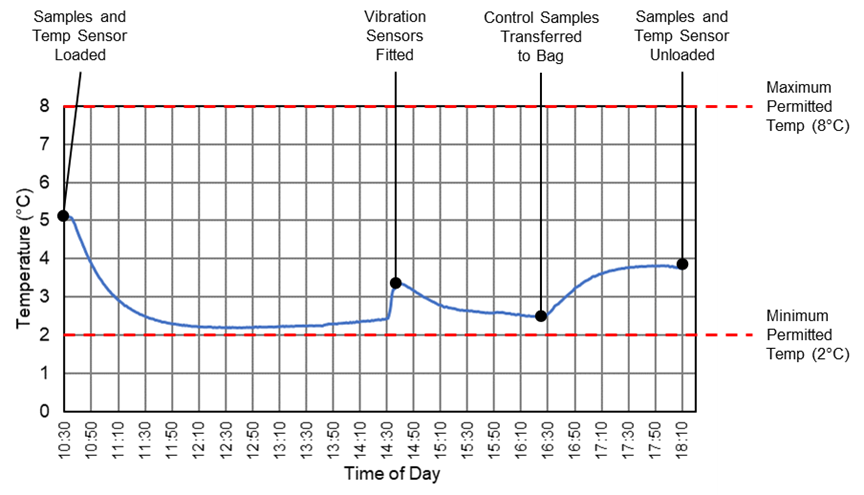
*

***S4 Figure.*** *Temperature monitoring during the experiment. The decrease in temperature after 10:30 occurred after loading.*
