# Supplementary material for: Investigating the influence of drone flight on the stability of cancer medicines
Source: PLoS One. 2023 Jan 6;18(1):e0278873. doi: 10.1371/journal.pone.0278873 (PMC9821719; doi:10.1371/journal.pone.0278873)
Supplement: S2 Fig — (a) Bevacizumab, (b) Trastuzumab, (c) Rituximab vibration (vortex) structural integrity determined by SE-HPLC. Data are shown as amounts of HMWS or LMWS (expressed in% of the main peak) ± SD (n = 3). (DOCX) [file pone.0278873.s006.docx]

| 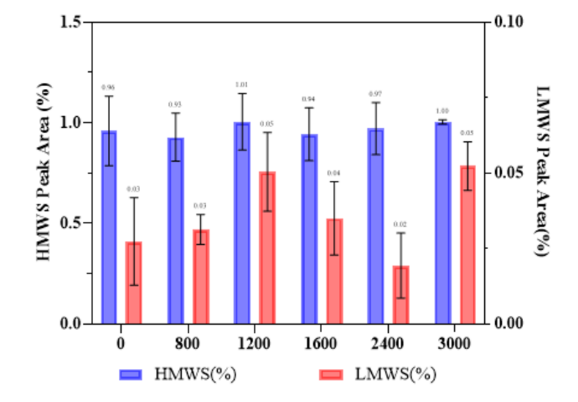(a) |
| --- |
| 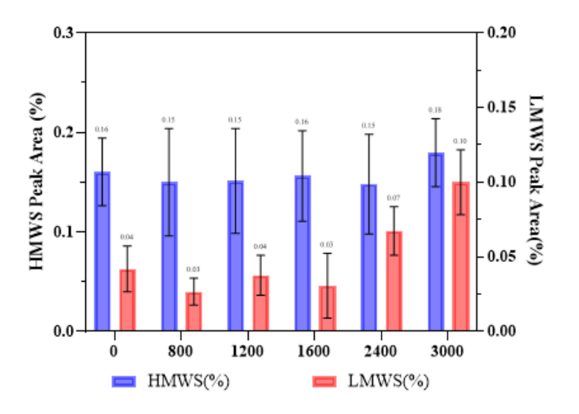(b)  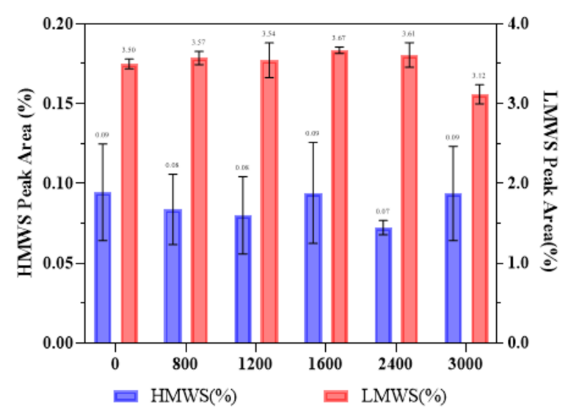(c) |

**S6 Figure.** (a) Bevacizumab, (b) Trastuzumab, (c) Rituximab vibration (vortex) structural integrity determined by SE-HPLC. Data are shown as amounts of HMWS or LMWS (expressed in% of the main peak) ± SD (n=3).
